# Poisoning in Ruminants by *Palicourea* Aubl. Species (Rubiaceae) in Brazil: A Review

**DOI:** 10.3390/vetsci12060540

**Published:** 2025-06-02

**Authors:** Flávia Aparecida de Oliveira Bezerra, Emily Rodrigues de Andrade, José Jailson Lima Bezerra, Antonio Fernando Morais de Oliveira

**Affiliations:** Departamento de Botânica, Universidade Federal de Pernambuco, Av. da Engenharia, s/n, Cidade Universitária, Recife 50670-420, PE, Brazil; fvoliveira232@gmail.com (F.A.d.O.B.); emily.era@ufpe.br (E.R.d.A.); afmoliveira@gmail.com (A.F.M.d.O.)

**Keywords:** livestock, *Palicourea marcgravii*, poisonous plants, sudden death, sodium monofluoroacetate

## Abstract

Some species belonging to the genus *Palicourea* (Rubiaceae) are known to cause poisoning in production animals on Brazilian rural properties, being responsible for negatively affecting the livestock sector of the country. In this context, the present study aimed to review reports of spontaneous and experimental poisoning in ruminants caused by *Palicourea* species in different regions of Brazil. The documents were retrieved from various databases, covering the period from the first report published in 1986 to April 2025. *Palicourea aeneofusca*, *Palicourea colorata* (Syn. *Psychotria colorata*), *Palicourea grandiflora*, *Palicourea hoffmannseggiana* (Syn. *Psychotria hoffmannseggiana*), *Palicourea marcgravii*, and *Palicourea violacea* (Syn. *Psychotria capitata*) have been identified as causes of spontaneous poisoning in the following Brazilian states: Pernambuco, Sergipe, Paraíba, Goiás, São Paulo, Minas Gerais, Tocantins, Rondônia, and Distrito Federal. In general, *Palicourea* species cause sudden death in poisoned animals, especially *P. marcgravii*. Although cattle are more susceptible to poisoning by these plants, there are reports of cases in sheep, goats, and buffalo. Sodium monofluoroacetate occurs in *Palicourea* species and has been well reported as the main compound responsible for cases of poisoning in these animals. Despite epidemiological evidence of spontaneous poisoning cases in Brazil, the species *P. colorata*, *P. grandiflora*, *P. hoffmannseggiana*, and *P. violacea* have not yet been experimentally tested for their toxic potential, and studies of this nature are necessary.

## Simple Summary

When animals such as cattle, sheep, and goats eat certain plants from the genus *Palicourea* (a group of plants belonging to the Rubiaceae family), it can be deadly for them. This is a big problem for farmers in Brazil, as these plants cause serious illness and sudden death in their livestock. These plants often cause rapid death in animals after eating them, with “erva-de-rato” (*Palicourea marcgravii*) being particularly dangerous. Although cattle are most commonly affected, other ruminants, such as sheep and goats, can also be poisoned. The main culprit behind these toxic effects is a chemical called sodium monofluoroacetate, which is found naturally in these plants and is highly poisonous to animals. Although we have strong evidence from real-life cases showing how dangerous these plants are, some species have not been thoroughly tested in controlled scientific experiments. More research is needed to fully confirm their toxic potential. This will help us better understand the risks and develop strategies to prevent future livestock poisonings.

## 1. Introduction

Poisonous plants of interest to livestock are those that, when consumed by production animals in natural conditions, can cause health problems or even lead to death [1]. Plant poisoning is considered one of the main causes of disease in livestock worldwide [2]. The occurrence of plant poisoning in production animals is recognized as one of the main causes linked to losses in livestock farming in several regions of Brazil [3,4,5]. The diagnosis of these poisonings is generally based on clinical signs, histopathological examinations, and the identification of the toxic compound in the plant [6].

The Rubiaceae Juss. family is well known for grouping together poisonous species that interfere with cardiac function and can cause the death of farm animals [7,8,9]. This family is composed of more than 13,000 species distributed in approximately 611 genera, occupying a wide range of tropical and subtropical ecosystems [10]. The genus *Palicourea* Aubl. presents a great diversity of alkaloids derived from tryptamine and its precursor, tryptophan, as well as monoterpene indole alkaloids [11,12]. In general, alkaloids are identified as important biomarkers in plants that cause poisoning in production animals [13]. In Brazil, *Palicourea* species containing sodium monofluoroacetate (MFA) are associated with several outbreaks of poisoning, mainly in cattle, although they occasionally also affect sheep, buffalo, and goats [7].

Due to its wide distribution in pasture areas and negative impact on livestock, *Palicourea marcgravii* (A.St.-Hil.) is widely recognized as the main poisonous plant in Brazil [14,15]. This species causes rapid and fatal effects, such as tachycardia, muscle tremors, and sudden death in ruminants, making early clinical intervention difficult and worsening animal losses [16]. It is important to emphasize that the diagnosis of sudden death in ruminants is not simple and must be based on careful observation of the remaining animals for acute clinical signs and on the necropsy of the deceased animal [17]. MFA is the main toxic compound identified in *P. marcgravii* and is responsible for causing sudden deaths in production animals after consumption of the plant [2,16,18,19]. This potent toxin blocks ATP production via the Krebs cycle and causes acute toxicity in ruminants that consume MFA-containing plants [20].

In general, studies on poisonous plants are essential to diagnose possible outbreaks, reduce economic losses, and identify the compounds responsible for poisoning [21]. Obtaining this data is of fundamental importance to implement effective measures against poisonous plants that occur on farms and negatively affect the livestock sector. In this context, the present study aimed to review reports of spontaneous and experimental poisoning in ruminants caused by *Palicourea* species in different regions of Brazil.

## 2. Methodology

### 2.1. Databases

The documents were retrieved from the Google Scholar (https://scholar.google.com/ (accessed on 30 May 2025)), PubMed^®^ (https://pubmed.ncbi.nlm.nih.gov/ (accessed on 30 May 2025)), Scopus (http://www.scopus.com/ (accessed on 30 May 2025)), (https://www.sciencedirect.com/search (accessed on 30 May 2025)), SciELO (https://search.scielo.org/ (accessed on 30 May 2025)), and Web of Science (https://www.webofknowledge.com (accessed on 30 May 2025)) databases. The keywords used in the article searches were “*Palicourea* AND poisoning AND Brazil”, “*Palicourea* AND toxicity”, “*Palicourea* AND ruminants”, “*Palicourea* AND natural poisoning”, “*Palicourea* AND experimental poisoning”, “*Palicourea* AND spontaneous poisoning”, “*Palicourea* AND fluoroacetate”, and “*Palicourea* AND monofluoroacetate”.

### 2.2. Inclusion and Exclusion Criteria

As inclusion criteria, articles with specific information on spontaneous and experimental poisonings in ruminants caused by *Palicourea* species in different regions of Brazil were selected from the first report published by Tokarnia et al. [22] in 1986 to April 2025. It is important to highlight that, during searches in the databases, some records published before 1986 on the topic were found; however, we were unable to retrieve the full text of these documents. Regarding the exclusion criteria, review articles, e-books, book chapters, undergraduate thesis, Master’ thesis, PhD thesis, and works published in technical or scientific events were excluded [5]. All scientific names of the species were checked on the platform The World Flora Online (WFO) Plant List (https://wfoplantlist.org/ (accessed on 30 May 2025)).

### 2.3. Data Screening and Information Categorization

**Figure 1:**
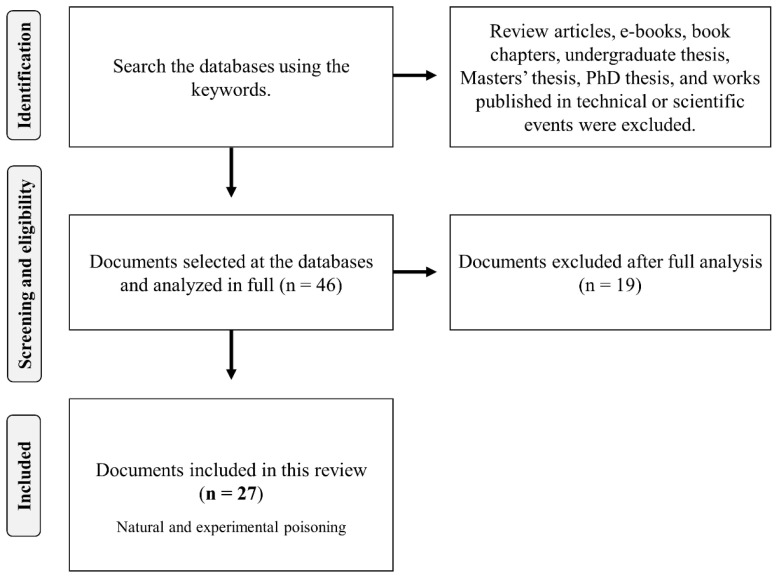
Flow diagram of selection of scientific documents included in this review.

A total of 46 scientific articles were selected from the databases (Figure 1). Subsequently, 19 of these studies were excluded because they presented information on the toxic potential of *Palicourea* in other animal species (non-ruminant), addressed only the chemical composition of the plants, or were review articles. Finally, 27 articles containing data on spontaneous poisonings (n = 14), experimental poisonings (n = 12), and both cases of poisonings in ruminants (n = 1) caused by *Palicourea* species were considered and included in the present study (Table S1—Supplementary Material). The collected data were presented in tables and figures when necessary. The results were described in three categories: (1) “*Spontaneous poisoning by Palicourea species*”, (2) “*Experimental poisoning by Palicourea species*”, and (3) “*Toxicity of sodium monofluoroacetate*”.

### 2.4. Statistical Analysis

Studies that address information on spontaneous poisonings in ruminants caused by *Palicourea* species in different regions of Brazil were statistically analyzed by relative frequency (RF), according to the formula of Sadat-Hosseini et al. [23] with modifications. The RF was obtained by dividing the number of documents per state (ND) by the total number of documents (N), according to the following formula: RF = (ND/N) × 100.

## 3. Results

### 3.1. Spontaneous Poisoning by Palicourea Species

**Figure 2:**
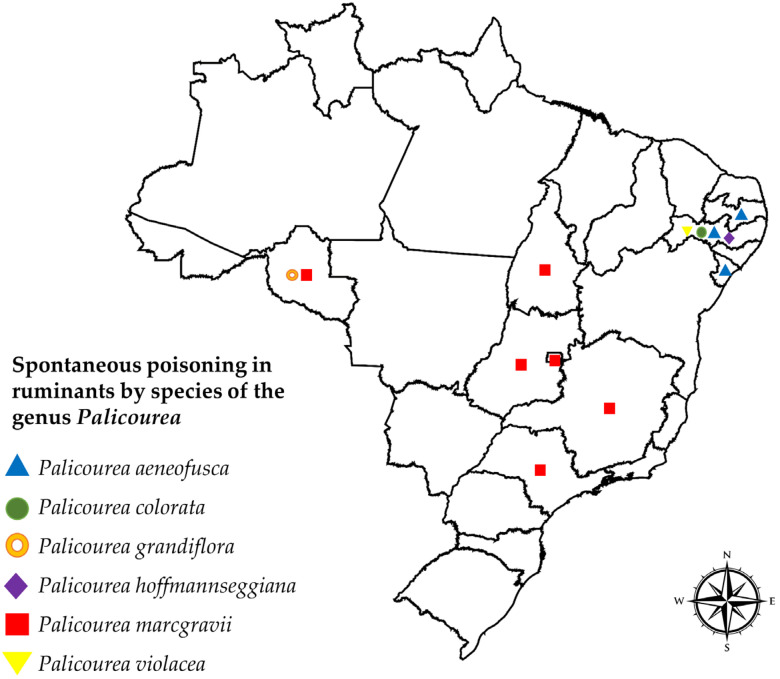
Geographic distribution of ruminant poisonings caused by *Palicourea* species in Brazil. Map produced by José Jailson Lima Bezerra in MapChart^©^ (https://mapchart.net, accessed on 6 December 2024).

Spontaneous poisoning in ruminants caused by *Palicourea* species have been reported in nine Brazilian states, mainly in Pernambuco (14.29%), Paraíba (14.29%), Goiás (14.29%), São Paulo (14.29%), and Tocantins (14.29%) (Table 1, Figure 2). It is important to highlight that no cases of poisoning were recorded in states in the South region of Brazil. Poisonous species of this genus, such as *Palicourea aeneofusca* (Müll.Arg.) Standl., *Palicourea colorata* (Hoffmanns. ex Willd.) Delprete & J.H.Kirkbr. (Syn. *Psychotria colorata*), *Palicourea grandiflora* (Kunth) Standl., *Palicourea hoffmannseggiana* (Willd. ex Schult.) Borhidi (Syn. *Psychotria hoffmannseggiana*), *Palicourea marcgravii* (A.St.-Hil.), and *Palicourea violacea* (Aubl.) A.Rich. (Syn. *Psychotria capitata*) have been reported from these regions. Cattle were the main animals affected; however, there are records that these plants also caused poisoning in sheep and goats (Table 2).

**Figure 3:**
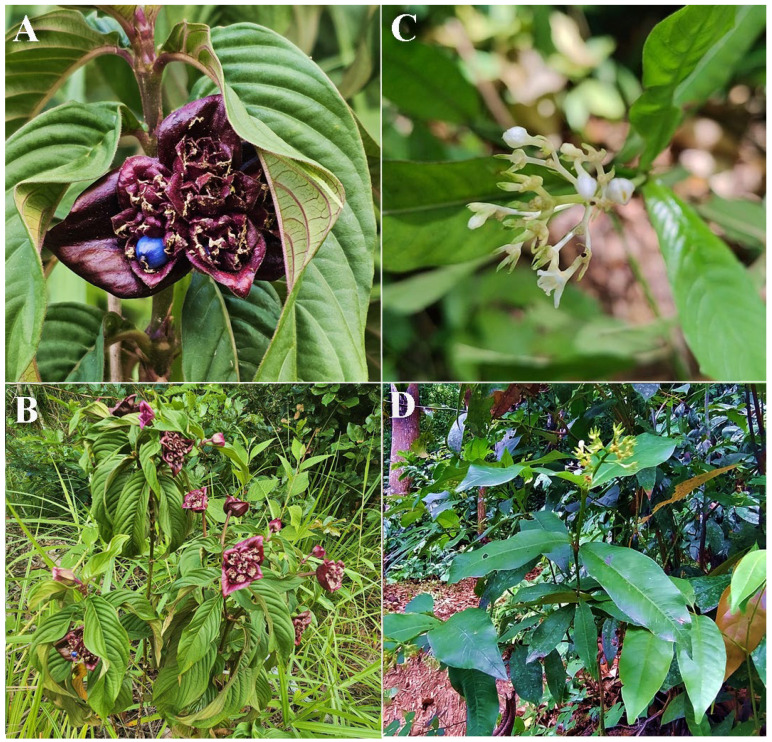
Natural occurrence of species of the genus *Palicourea* in the Atlantic Forest of Recife, state of Pernambuco, Brazil. (**A**,**B**) *Palicourea colorata*. (**C**,**D**) *Palicourea violacea*. Photos by Flavia Oliveira.

Cases of poisoning by several species of *Palicourea* (Figure 3) in production animals have been well reported in the Northeast region of Brazil [21,24,25,26,27]. In the dairy region of Pernambuco, *P. colorata* (Syn. *Psychotria colorata*), *P. hoffmannseggiana* (Syn. *Psychotria hoffmannseggiana*), and *P. violacea* (Syn. *Psychotria capitata*) have been reported as poisonous to cattle, but scientific information to support this hypothesis is lacking [27]. The clinical signs observed in poisoned animals included gait instability, muscle tremors, and deaths after the cattle were forced to move. It is important to highlight that, to date, no scientific studies have been found that prove the toxic effects of *P. colorata*, *P. hoffmannseggiana*, and *P. violacea*, and phytochemical and toxicological investigations are necessary to determine the possible mechanisms of action involved in the deaths of the animals.

*Palicourea marcgravii* has been reported as causing poisoning in cattle, goats, and sheep in several states in the Midwest, North, and Southeast regions of Brazil [9,15,19,28,29,30]. The clinical signs observed in animals poisoned by *P. marcgravii* were described with the following characteristics: urination, ataxia, agitation, lateral recumbency, muscle tremors, pedaling movements, dyspnea, loss of balance, depression, falls, convulsions, polyuria, engorged jugular veins, tachycardia, arrhythmia, increased respiratory rate, and sudden death (Table 2).

Some studies reported difficulties related to prophylaxis and control of *P. marcgravii* on rural properties to avoid new cases of poisoning [9,15,28]. Paim et al. [15] reported that one of the factors that make prophylaxis difficult is the depth of the root of *P. marcgravii*; even when pulled out by producers, the plant grows back. Therefore, the best option is to inspect pastures to identify the occurrence of this plant and fence the areas to prevent cattle access [9]. Another solution is to stimulate the development of tolerance and resistance of animals to poisoning by *P. marcgravii* [28]. Costa et al. [2] reported that the administration of sodium trifluoroacetate in cattle can induce effective resistance against poisoning by plants containing sodium monofluoroacetate. It is important to highlight that each rural property has different characteristics, and monitoring by a qualified professional in the area is essential to determine and execute the necessary prophylactic measures.

**Table 2:** Spontaneous poisoning in ruminants caused by *Palicourea* species in different regions of Brazil.

Species	Affected Animals	Clinical Signs	Outbreak Year	Brazilian State	References
*Palicourea aeneofusca* (Müll. Arg.) Standl.	Cattle	Sudden death	Not specified	Pernambuco	Melo et al. [27]
	Cattle	Sudden death associated with exercise	2015 and 2016	Sergipe	Nascimento et al. [21]
	Cattle	Lethargy, sternal recumbency, lateral recumbency, fatigue, tachypnea, tachycardia, muscle tremors, pedaling movements, and death	2012 and 2013	Pernambuco	Brito et al. [25]
	Cattle	Muscle tremors, falls, and death	2007	Paraíba	Vasconcelos et al. [24]
	Goats	Motor incoordination, muscle tremors, tachypnea, tachycardia, dyspnea, pedaling movements, and death	2016	Paraíba	Oliveira Neto et al. [26]
*Palicourea colorata* (Hoffmanns. ex Willd.) Delprete & JHKirkbr. (Syn. *Psychotria colorata*)	Cattle	Gait instability, muscle tremors, and death	Not specified	Pernambuco	Melo et al. [27]
*Palicourea grandiflora* (Kunth) Standl.	Sheep	Muscle tremors, falls, and death	2008 to 2011	Rondônia	Schons et al. [31]
*Palicourea hoffmannseggiana* (Willd. ex Schult.) Borhidi (Syn. *Psychotria hoffmannseggiana)*	Cattle	Gait instability, muscle tremors, and death	Not specified	Pernambuco	Melo et al. [27]
*Palicourea marcgravii* (A.St.-Hil.)	Cattle	Sudden death	2008 to 2011	Rondônia	Schons et al. [31]
	Cattle	Lateral recumbency, pedaling movements, opisthotonus, jugular engorgement, and death	2010 and 2012	Goiás	Sant’Ana et al. [32]
	Cattle	Urination, ataxia, and death	Not specified	Goiás	Paim et al. [15]
	Sheep	Agitation, isolation, lateral recumbency, muscle tremors, pedaling movements, and death	Not specified	São Paulo	Koether et al. [19]
	Cattle	Dyspnea, loss of balance, muscle tremors, lateral recumbency, pedaling movements, and death	1953 to 2018	Southeast, Midwest, and North Region	Ubiali et al. [9]
	Cattle	Depression, falls, ataxia, seizures, polyuria, and death	2007 and 2008	Tocantins	Costa et al. [29]
	Cattle	Sudden death	Not specified	Distrito Federal	Ferreira Junior et al. [33]
	Cattle	Muscle tremors, engorged jugular veins, falling, pedaling movements, tachycardia, arrhythmia, dyspnea, and death	2010 and 2011	Tocantins	Helayel et al. [30]
	Cattle, goats, and sheep	Muscle tremors, seizures, and death	2000	São Paulo	Soto-Blanco et al. [28]
	Cattle	Falls, lateral recumbency, pedaling movements, opisthotonus, jugular engorgement, and death	Not specified	Minas Gerais	Alves et al. [34]
*Palicourea violacea* (Aubl.) A.Rich. (Syn. *Psychotria capitata*)	Cattle	Gait instability, muscle tremors, and death	Not specified	Pernambuco	Melo et al. [27]

### 3.2. Experimental Poisoning by Palicourea Species

A total of six *Palicourea* species have been reported as causing spontaneous poisonings in Brazil (Table 2); however, only three have been investigated through experimental studies with ruminants. These experimental studies investigated the toxic potential of *P. juruana* [35,36], *P. aeneofusca* [18,25,37], and *P. marcgravii* [2,16,38,39,40,41,42,43] in cattle, goats, sheep, and buffalo. The summary of these results is described in Table 3.

#### 3.2.1. *Palicourea juruana* K.Krause.

*Palicourea juruana* has been the target of research to evaluate its toxic effects on cattle and buffalo [35,36]. Consuming fresh leaves of this plant can result in different symptoms depending on the dose administered. According to Oliveira et al. [35], when 2 g/kg of fresh leaves was ingested by a buffalo, the animal presented symptoms such as sternal recumbency, pedaling movements, opisthotonus, dyspnea, and death. In cattle, ingestion of 0.25 g/kg of fresh leaves caused imbalance, falling, lateral recumbency, and death. Histopathological findings revealed the presence of lesions in the liver and myocardium. The poisoning by *P. juruana* in buffaloes and cattle demonstrated that, although buffaloes are more resistant to the toxin, with lethal doses between 1 and 2 g/kg, the clinical signs observed in both species were similar [35]. Santos et al. [36] reported that cattle previously poisoned by *P. juruana* presented the following alterations in the heart: marked coagulative necrosis of cardiomyocytes with loss of striations and areas of fiber flocculation, associated with a marked inflammatory infiltrate composed mainly of macrophages.

#### 3.2.2. *Palicourea aeneofusca* (Müll. Arg.) Standl.

Experimental poisoning by *P. aeneofusca* has been observed in cattle and goats [18,25,37]. According to Oliveira et al. [37], the administration of 0.02 and 0.06 g/kg of dehydrated leaves of *P. aeneofusca* in goats resulted in falls, apathy, anorexia, tachycardia, imbalance, motor incoordination, lateral recumbency, opisthotonus, nystagmus, pedaling movements, and death. Strategies related to the induction of conditioned aversion to this plant were also investigated. Oliveira et al. [18] reported that the administration of 0.35 g/kg of fresh leaves of *P. aeneofusca* followed by 175 mg/kg of lithium chloride (LiCl) was effective in inducing conditioned food aversion in goats, without causing toxicity or clinical symptoms. The aversion persisted for 90 days, even with the high palatability of the plant. For cattle, aversion to *P. aeneofusca* induced by a single dose of LiCl persisted for 12 months [25]. This approach may be promising for preventing plant poisoning in goats and cattle.

#### 3.2.3. *Palicourea marcgravii* (A.St.-Hil.)

Popularly known as “erva-de-rato”, *P. marcgravii* stands out as one of the main poisonous plants in Brazil. Cattle are the main animals affected; however, there are records of experimental poisoning in goats, buffaloes, and sheep [2,16,38,39,40,41,42,43]. According to Barbosa et al. [38], cattle that received a dose of 2 g/kg of fresh leaves of *P. marcgravii* showed moderate hydropic-vacuolar degeneration of the epithelial cells of the distal convoluted tubules of the kidneys in histopathological examinations. Similar results were observed in buffaloes poisoned with 4 g/kg of fresh leaves of this plant [38].

Histopathological examinations of cattle poisoned with 1.0 g/kg of fresh leaves of *P. marcgravii* revealed the presence of individual muscle fibers or groups of cardiomyocytes with increased sarcoplasmic eosinophilia, loss of striations, and, sometimes, pyknotic nuclei in the hearts of the animals [40]. In Nelore, Curraleiro Pé-Duro, and Pantaneiro calves that received a dose of 1.8 g/kg of the leaves of this plant, severe histopathological lesions were observed in the kidneys, including multifocal cytoplasmic macro-vacuolization in epithelial cells of the convoluted tubules and in some medullary tubules, with marked nuclear pyknosis [41].

In sheep experimentally poisoned by *P. marcgravii*, it was found through electrocardiographic and echocardiographic examinations that the cause of death in these animals was acute heart failure characterized by arrhythmias, ventricular tachycardia/fibrillation, a drop in cardiac output, left ventricular systolic dysfunction, decreased fractional shortening of the left ventricle, and decreased aortic flow velocity and aortic flow gradient [16]. In another study, it was observed that the myocardium of sheep that received 1 g/kg of *P. marcgravii* presented areas and foci of coagulative necrosis, characterized by an increase in the cytoplasm of muscle fibers and pyknosis [42]. These results demonstrate the severity of poisoning by this plant in sheep and reinforce the importance of adopting prophylactic measures against *P. marcgravii* on rural properties.

**Table 3:** Experimental poisonings in ruminants by *Palicourea* species.

Species	Part of the Plant	Dose	Animals	Clinical Signs	References
*Palicourea juruana* K. Krause	Fresh leaves	0.25–2 g/kg	Buffaloes	Lateral recumbency, sternal recumbency, disordered movements, opisthotonus, dyspnea, and death	Oliveira et al. [35]
	Fresh leaves	0.125–2 g/kg	Cattle	Falls, imbalance, sternal recumbency, pedaling movements, lateral recumbency, opisthotonus, dyspnea, death	Oliveira et al. [35]
*Palicourea aeneofusca* (Müll. Arg.) Standl.	Fresh leaves	35 mg/kg	Cattle	On the fifth day of the experiment, two cattle showed signs of poisoning and died	Brito et al. [25]
	Fresh leaves	0.35 g/kg	Goats	Lithium chloride administration in goats was effective in inducing conditioned food aversion to *P. aeneofusca*	Oliveira et al. [18]
	Dehydrated leaves	0.02–0.06 g/kg	Goats	Falls, apathy, anorexia, tachycardia, imbalance, motor incoordination, lateral recumbency, opisthotonus, nystagmus, pedaling movements, and death	Oliveira et al. [37]
*Palicourea marcgravii* (A. St.-Hil.)	Fresh and dehydrated plant	0.5–1 g/kg	Sheep	Tachypnea, tachycardia, sternal recumbency, difficulty walking, muscle tremors, lateral recumbency, pedaling movements, opisthotonus, and death	Tokarnia et al. [22]
	Fresh leaves	0.25–2.0 g/kg	Cattle	Sternal recumbency, lateral recumbency, tachycardia, engorged jugular vein, disordered movements, dyspnea, and death	Barbosa et al. [38]
	Fresh leaves	0.5–6.0 g/kg	Buffaloes	Muscle tremors, sternal recumbency, lateral recumbency, pedaling movements, engorged jugular vein, dyspnea, opisthotonus, and death	Barbosa et al. [38]
	Fresh leaves	1.0 g/kg	Cattle	Tachycardia, tachypnea, muscle tremors, pollakiuria, instability, loss of balance, lateral recumbency, pedaling movements, dyspnea, arrhythmia, opisthotonus, nystagmus, and death	Peixoto et al. [40]
	Fresh leaves	0.2 and 2 g/kg	Goats	Tachycardia, anorexia, apathy, arrhythmia, tachypnea, sternal recumbency, and death	Barbosa et al. [39]
	Fresh leaves	2.0 g/kg	Calves	Jugular vein distension, reluctance to exercise, weakness, motor incoordination, ataxia, and sternal recumbency	Costa et al. [2]
	Leaves	1.8 g/kg	Cattle	Falls, apathy, muscle tremors, loss of appetite, jugular distension, tachycardia, abdominal breathing, lateral recumbency, sternal recumbency, pedaling movements, and death	Serodio et al. [41]
	Fresh plant	1 g/kg	Sheep	Abdominal breathing, coughing, head pressure, and nystagmus	Cunha et al. [42]
	Fresh plant	1 g/kg	Sheep	Deaths caused by acute heart failure, arrhythmias, ventricular tachycardia/fibrillation, and decreased cardiac output	Cunha et al. [16]
	Fresh plant	1 g/kg	Sheep	Cardiac lesions detected by analysis of serum troponin I levels	Cunha et al. [43]

### 3.3. Toxicity of Sodium Monofluoroacetate

**Figure 4:**
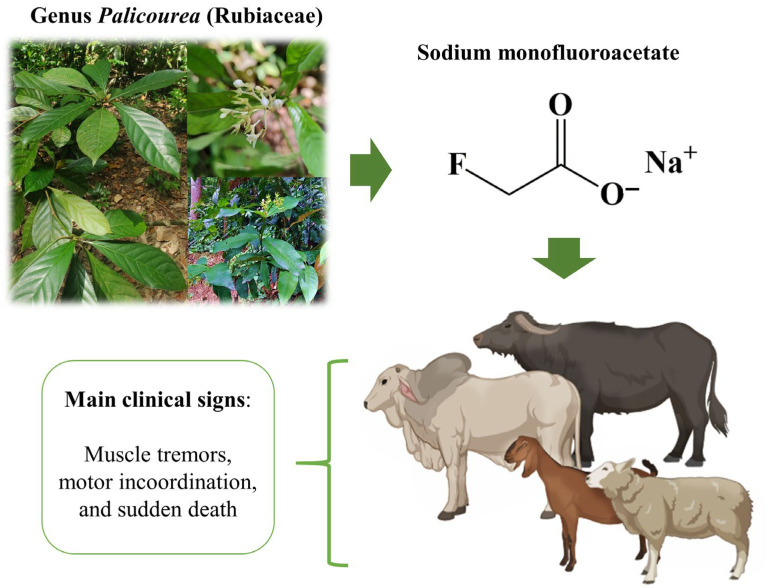
Illustration of poisoning in ruminants by *Palicourea* species containing sodium monofluoroacetate. Created in https://biorender.com, accessed on 6 December 2024.

In Brazil, the numerous plants that cause sudden death are responsible for significant economic losses, with an estimated loss of at least 600,000 head of cattle per year [44]. Among these plants, those belonging to the *Palicourea* genus stand out, which cause sudden deaths associated with exercise and contain high concentrations of sodium monofluoroacetate (Figure 4) [7]. In addition to sudden death, MFA is also associated with cases of reproductive disorders in ruminants [45,46]. Although this compound was discovered around 1896, in Brazil, MFA was first identified in dehydrated leaves of *P. marcgravii* using the column chromatography method [47]. Lee et al. [48] also reported the occurrence of MFA in *P. marcgravii* and *P. aeneofusca* at concentrations of 0.24% and 0.09%, respectively. The high concentration of MFA in *P. marcgravii* may be directly related to the large number of outbreaks of poisoning in ruminants in different regions of Brazil. In a later study, Cook et al. [49] identified the occurrence of MFA through the HPLC–APCI–MS method in several other *Palicourea* species, including *P. grandiflora*, *P.* aff. *juruana*, *P. amapaensis*, *P. longiflora*, *P.* aff. *longiflora*, *P. macarthurorum*, *P. nigricans*, and *P. vacillans*.

In an experiment conducted by Nogueira et al. [50], it was reported that doses of 0.5 and 1.0 mg/kg of MFA administered orally to cattle caused lesions characterized by hydropic-vacuolar degeneration of the distal convoluted uriniferous tubules and, eventually, of the collecting tubules, associated with karyopyknosis. Regarding the mode of action of MFA, it has been described that fluoroacetate binds to acetyl coenzyme A (CoA) to form fluoroacetyl CoA, which replaces acetyl CoA in the Krebs cycle and reacts with citrate synthase to produce fluorocitrate. This toxic metabolite blocks aconitase, preventing the formation of the coenzymes NADH and FADH_2_, and consequently, there is no transfer of electrons to the respiratory chain and formation of ATP from ADP, culminating in the blockage of the Krebs cycle [51].

## 4. Discussion

Many studies that reported the occurrence of spontaneous poisoning in ruminants by *Palicourea* species in different regions of Brazil did not specify the period of the outbreaks on rural properties (Table 2). These gaps may hinder the development of future studies, as the wealth of detail in epidemiological data is very important for monitoring over time and strategic planning to prevent new outbreaks of poisoning. Furthermore, there is another major problem related to the correct identification of the scientific names of plants that cause poisoning in animals. In some studies, the authors included synonyms instead of the accepted scientific names of plants. For example, species belonging to the genus *Psychotria* are often confused with species of *Palicourea*. These limitations in the content of articles in the area of poisonous plants reinforce the need for knowledge from botanical taxonomists to assist in the correct identification of plants.

Although *Palicourea* species occur in all geographic regions of Brazil, these plants are also commonly found in other countries, including Mexico, Peru, Bolivia, Colombia, Ecuador, Paraguay, and Argentina [52,53]. However, no records were found of cases of poisoning by *Palicourea* species in ruminants in these other countries in Central and South America. Considering the well-documented cases of poisoning in Brazil, it is important to disseminate this knowledge so that producers in other regions of the world where these plants occur can adopt preventive measures to avoid animal losses due to poisoning on their rural properties.

Just as the *Palicourea* genus is well known for causing economic losses on several Brazilian properties, other cardiotoxic plants are also associated with cases of sudden death in ruminants in the country [7]. Several studies have reported that *Ateleia glazioveana* Baill. [54,55], *Nerium oleander* L. [56,57], *Amorimia septentrionalis* W.R. Anderson [58], and *Niedenzuella stannea* (Griseb.) W.R. Anderson [59,60,61] affect cardiac function and cause death in poisoned animals.

*Heterophyllaea pustulata* Hook.f. is another species belonging to the Ruabiaceae family reported as causing poisoning in ruminants in Argentina [62,63]. According to Micheloud et al. [62], sheep poisoned by this species showed clinical signs of photosensitization, including photophobia, itching, restlessness, edema on the face and ears, intense tearing, and necrosis with formation of crusts on the skin. These authors associated photosensitization of animals with the ingestion of anthraquinones present in the leaves of *H. pustulata*. In addition to sheep, Micheloud et al. [63] also reported that goats that ingested the leaves of this plant presented photophobia, pruritus, restlessness, congestion, edema, anorexia, and blindness. Histopathological findings of the liver revealed diffusely and moderately swollen hepatocytes and finely granular cytoplasm with irregular cell borders [63].

Sodium monofluoroacetate was also identified in other species belonging to the Malpighiaceae and Bignoniaceae families that cause poisoning in ruminants in Brazil [59,64,65,66]. According to Cunha et al. [64], MFA was isolated from *Mascagnia rigida* (*Amorimia rigida* (A. Juss.) W.R. Anderson) using chromatography and infrared spectroscopy techniques. Lima et al. [65] detected the occurrence of MFA in the leaves of *Amorimia pubiflora* (A. Juss.) W.R.Anderson using the HPLC method. Species of the genus *Amorimia* generally present MFA concentrations ranging from 0.0007 to 0.02% [48]. *Niedenzuella stannea* (Griseb.) W.R. Anderson, a species reported to cause sudden death in cattle, showed different MFA concentrations depending on the plant part analyzed by HPLC–APCI–MS (0.06–0.0003%) [59]. These concentrations are lower than those found for *Palicourea* species, where *P. aeneofusca* and *P. marcgravii* present concentrations ranging from 0.09 to 0.24% MFA [48].

In addition to MFA, other chemical compounds belonging to the alkaloid class have already been identified in *Palicourea* species that are mentioned as toxic to ruminants. These compounds were identified in *P. marcgravii* [67,68,69] and *P. hoffmannseggiana* (Syn. *Psychotria barbiflora*) [69,70]. However, it is important to highlight that no records were found on the relationship between these substances and cases of poisoning. In this context, it is likely that MFA is, in fact, responsible for animal deaths associated with the consumption of Palicourea species in different regions of Brazil.

## 5. Conclusions

Spontaneous poisonings in ruminants caused by *P. aeneofusca*, *P. colorata* (Syn. *Psychotria colorata*), *P. grandiflora*, *P. hoffmannseggiana* (Syn. *Psychotria hoffmannseggiana*), *P. marcgravii*, and *P. violacea* (Syn. *Psychotria capitata*) were recorded in the Northeast, North, Midwest, and Southeast regions of Brazil. *Palicourea* species generally cause sudden death in poisoned animals, especially *P. marcgravii*. Although cattle are more susceptible to poisoning by these plants, there are reports of cases in sheep, goats, and buffalo. Sodium monofluoroacetate has been widely identified in *P. marcgravii*, and several studies have highlighted that this is the main compound responsible for poisoning cases in farm animals.

## 6. Future Directions

Despite epidemiological evidence of cases of spontaneous poisoning in Brazil, the species *P. colorata*, *P. grandiflora*, *P. hoffmannseggiana*, and *P. violacea* have not yet been experimentally tested for their toxic potential, and studies of this nature are necessary. Considering that these plants belong to the genus *Palicourea*, it is likely that their toxic active component is also sodium monofluoroacetate. However, it is necessary to carry out phytochemical analyses of the extracts obtained from these species to confirm the occurrence of this compound and identify other possible toxic substances, such as alkaloids, which may also be associated with cases of poisoning in ruminants.

Taken together, this information reinforces the importance of adopting effective prophylactic measures in ruminant production areas to prevent contact between animals and plants of the *Palicourea* genus. As reported in previous studies, measures such as pruning leaves and branches of toxic species, using herbicides to control poisonous plants, fencing off infested areas, and inspecting the forage offered to animals can be effective alternatives to reduce losses caused by plant poisoning.

## Figures and Tables

**Table 1:** Number of scientific articles with reports of spontaneous poisoning in ruminants caused by *Palicourea* species by geographic region and state of Brazil.

Geographic Region	Brazilian State *	DN	RF (%)
Northeast	Pernambuco (PE)	2	14.29
	Paraíba (PB)	2	14.29
	Sergipe (SE)	1	7.14
Midwest	Goiás (GO)	2	14.29
	Distrito Federal (DF)	1	7.14
North	Tocantins (TO)	2	14.29
	Rondônia (RO)	1	7.14
Southeast	São Paulo (SP)	2	14.29
	Minas Gerais (MG)	1	7.14
Total		14	100

* Scientific documents that did not specify the Brazilian state where the research was carried out were not included in this relative frequency analysis. DN: document numbers per state; RF: relative frequency.

## Data Availability

The data used to support the findings of this study are included in this article.

## Supplementary Materials

The following supporting information can be downloaded at: https://www.mdpi.com/article/10.3390/vetsci12060540/s1, Table S1: Characterization of the articles selected in the databases and included in the review.

## Abbreviations

The following abbreviations are used in this manuscript:

ADP	Adenosine diphosphate
ATP	Adenosine triphosphate
CoA	Coenzyme A
HPLC–APCI–MS	High-performance liquid chromatography–atmospheric pressure chemical ionization–tandem mass spectrometry
MFA	Sodium monofluoroacetate
ND	Number of documents per state
N	Total number of documents
RF	Relative frequency
WFO	World Flora Online
