# Supplementary material for: Poisoning in Ruminants by Palicourea Aubl. Species (Rubiaceae) in Brazil: A Review
Source: Vet Sci. 2025 Jun 2;12(6):540. doi: 10.3390/vetsci12060540 (PMC12197330; doi:10.3390/vetsci12060540)
Supplement: Supplementary file 1 [file vetsci-12-00540-s001.zip › vetsci-3661267-supplementary.pdf]

Supplementary Material

Poisoning in ruminants by *Palicourea* Aubl. species (Rubiaceae) in Brazil: A review

Flávia Aparecida de Oliveira Bezerra, Emily Rodrigues de Andrade, José Jailson Lima Bezerra*, Antonio Fernando Morais de Oliveira

Universidade Federal de Pernambuco, Departamento de Botânica, Av. da Engenharia, s/n, Cidade Universitária, 50670-420, Recife, PE, Brazil

*Corresponding author. E-mail address: josejailson.bezerra@hotmail.com (J.J.L. Bezerra), Orcid ID: 0000-0003-2081-8304

Journal: *Veterinary Sciences*

Table S1. Characterization of the articles selected in the databases and included in the review (n = 27).

Databases	Title	Author
Google Scholar	Intoxicação experimental por Palicourea marcgravii (Rubiaceae) em ovinos	Tokarnia et al. [22]
	Intoxicação natural de caprinos e ovinos por *Palicourea marcgravii* St. Hil. (Rubiaceae).	Soto-Blanco et al. [28]
	Plantas tóxicas de interesse pecuário em região de Ecótono Amazônia e Cerrado. Parte II: Araguaína, norte do Tocantins.	Costa et al. [29]
	Intoxicação natural por *Palicourea marcgravii* (Rubiaceae) em bovinos no Estado do Tocantins.	Helayel et al. [30]
	Spontaneous poisoning by *Palicourea marcgravii* in bovine in the Federal District, Brazil.	Ferreira Junior et al. [33]
	Plantas tóxicas para bovinos em Minas Gerais.	Alves et al. [34]
SciELO	Comparação da sensibilidade de bovinos e búfalos à intoxicação por *Palicourea marcgravii* (Rubiaceae).	Barbosa et al. [38]
	Estudo comparativo da toxidez de Palicourea juruana (Rubiaceae) para búfalos e bovinos.	Oliveira et al. [35]
	Efeito protetor da acetamida em bovinos indica monofluoroacetato como princípio tóxico de *Palicourea marcgravii* (Rubiaceae).	Peixoto et al. [44]

Indução de resistência à intoxicação por *Palicourea aeneofusca* (Rubiaceae) mediante administração de doses sucessivas não tóxicas.	Oliveira et al. [37]
Conditioned food aversion to control *Palicourea aeneofusca* poisoning.	Oliveira et al. [39]
Sinais clínicos e patologia da intoxicação crônica experimental de caprinos por *Palicourea marcgravii.*	Barbosa et al. [39]
Poisonous plants for ruminants in the dairy region of Pernambuco, Northeastern Brazil.	Melo et al. [27]
Clinical and laboratory findings in sheep experimentally poisoned by *Palicourea marcgravii* and the use of magnesium chloride with possible therapeutic effect on poisoning.	Cunha et al. [42]
Electrocardiographic, echocardiographic and heart biomarker parameters in sheep experimentally poisoned by *Palicourea marcgravii* (Rubiaceae).	Cunha et al. [16]
Serum and immunohistochemical analyses of troponin I in sheep experimentally poisoned with *Palicourea marcgravii*.	Cunha et al. [43]
Plantas tóxicas para ruminantes e equídeos do estado de Sergipe.	Nascimento et al. [7]

	Spontaneous and experimental poisoning of cattle by *Palicourea aeneofusca* in the region of Pernambuco and induction of conditioned food aversion.	Brito et al. [25]
	Mortes súbitas em bovinos causadas por *Palicourea aeneofusca* (Rubiaceae) e *Mascagnia rigida* (Malpighiaceae) na Zona da Mata Paraibana.	Vasconcelos et al. [24]
	Intoxicações por plantas em ruminantes e equídeos na região central de Rondônia.	Schons et al. [31]
	Plantas tóxicas para ruminantes do Sudoeste de Goiás.	Sant'Ana et al. [32]
Scopus	Poisoning in goats by the monofluoracetate-containing plant *Palicourea aeneofusca* (Rubiaceae).	Oliveira Neto et al. [26]
	Sequential administrations of trifluoroacetate induce tolerance to *Palicourea maccgravii*, a monofluoroacetate-containing plant, in calves.	Costa et al. [2]
	Evaluation of the resistance of Nellore, Curraleiro Pe-duro and Pantaneiro cattle breeds by experimental intoxication of *Palicourea marcgravii*.	Serodio et al. [41]
	Toxic plants from the perspective of a "Quilombola" community in the Cerrado region of Brazil.	Paim et al. [15]

	Spontaneous poisoning by *Palicourea marcgravii* (Rubiaceae) in a sheep herd in southeastern Brazil.	Koether et al. [19]
PubMed	*Palicourea marcgravii* (Rubiaceae) poisoning in cattle grazing in Brazil.	Ubiali et al. [9]

References

2. Costa, A.G.; Carvalho, A.Ú.; Melo, M.M.; Soto-Blanco, B. Sequential administrations of trifluoroacetate induce tolerance to *Palicourea maccgravii*, a monofluoroacetate-containing plant, in calves. *Toxicon*. **2019**, *160*, 8-11. https://doi.org/10.1016/j.toxicon.2019.02.005

7. Nascimento, E.M.; Medeiros, R.M.; Riet-Correa, F. Plantas tóxicas para ruminantes e equídeos do estado de Sergipe. *Pesq. Vet. Bras*. **2018**, *38*, 835-839. https://doi.org/10.1590/1678-5150-PVB-5408

9. Ubiali, D.G.; Cardoso, L.F.C.; Pires, C.A.; Riet-Correa, F. *Palicourea marcgravii* (Rubiaceae) poisoning in cattle grazing in Brazil. *Trop. Anim. Health Prod*. **2020**, *52*, 3527-3535. https://doi.org/10.1007/s11250-020-02388-2

15. Paim, R.D.C.S.; Paula, L.G.F.; Soares, D.M.; Rocha, T.F.G.; Ribeiro, A.L.; Barros, N.; et al. Toxic plants from the perspective of a "Quilombola" community in the Cerrado region of Brazil. *Toxicon*. **2023**, *224*, 107028. https://doi.org/10.1016/j.toxicon.2023.107028

16. Cunha, I.M.; Lessa, D.A.; Carvalho, V.A.; Alencar, N.X.; Teixeira, A.L.; Chenard, M.G.; Souza, G.N.; Helayel, M.J.S. Electrocardiographic, echocardiographic and heart biomarker parameters in sheep experimentally poisoned by *Palicourea marcgravii* (Rubiaceae). *Pesq. Vet. Bras*. **2022**, *42*, e07097. https://doi.org/10.1590/1678-5150-PVB-7097

18. Oliveira, M.D.; Riet-Correa, F.; Silva, G.B.D.; Pereira, W.D.S.; Freire, L.F.S.; Medeiros, R.M.T. Conditioned food aversion to control *Palicourea aeneofusca* poisoning. *Cienc. Rural*. **2014**, *44*, 1246-1248. https://doi.org/10.1590/0103-8478cr20131369

19. Koether, K.; Lee, S.T.; Belluci, R.S.; Garcia, R.; Pfister, J.A.; Cunha, P.H.J.; Rocha, N.S.; Borges, A.S.; Oliveira-Filho, J.P. Spontaneous poisoning by *Palicourea marcgravii* (Rubiaceae) in a sheep herd in southeastern Brazil. *Toxicon*. **2019**, *161*, 1-3. https://doi.org/10.1016/j.toxicon.2019.02.015

22. Tokarnia, C.H.; Peixoto, P.V.; Döbereiner, J. Intoxicação experimental por *Palicourea marcgravii* (Rubiaceae) em ovinos. *Pesq. Vet. Bras*. **1986**, *6*, 121-131.

25. Brito, L.B.D.; Albuquerque, R.F.; Rocha, B.P.; Albuquerque, S.S.; Lee, S.T.; Medeiros, R.M.T.; Riet-Correa, F.; Mendonça, F.D.S. Spontaneous and experimental poisoning of cattle by *Palicourea aeneofusca* in the region of Pernambuco and induction of conditioned food aversion. *Cienc. Rural*. **2016**, *46*, 138-143. https://doi.org/10.1590/0103-8478cr20150079

24. Vasconcelos, J.S.D.; Riet-Correa, F.; Dantas, A.F.M.; Medeiros, R.M.; Dantas, Á.J.D.A. Mortes súbitas em bovinos causadas por *Palicourea aeneofusca* (Rubiaceae) e *Mascagnia rigida* (Malpighiaceae) na Zona da Mata Paraibana. *Pesq. Vet. Bras*. **2008**, *28*, 457-460. https://doi.org/10.1590/S0100-736X2008001000003

26. Oliveira Neto, T.S.; Riet-Correa, F.; Lee, S.T.; Cook, D.; Barbosa, F.M.S.; Silva Neto, J.F.; Simões, S.V.D.; Lucena, R.B. Poisoning in goats by the monofluoracetate-containing plant *Palicourea aeneofusca* (Rubiaceae). *Toxicon*. **2017**, *135*, 12-16. https://doi.org/10.1016/j.toxicon.2017.05.025

27. Melo, J.K.; Ramos, T.R.; Baptista Filho, L.C.; Cruz, L.V.; Wicpolt, N.S.; Fonseca, S.; Mendonça, F.S. Poisonous plants for ruminants in the dairy region of Pernambuco, Northeastern Brazil. *Pesq. Vet. Bras*. **2021**, *41*, e06807. https://doi.org/10.1590/1678-5150-PVB-6807

28. Soto-Blanco, B.; Haraguchi, M.; Silva, J.A.; Górniak, S.L. Intoxicação natural de caprinos e ovinos por *Palicourea marcgravii* St. Hil. (Rubiaceae). *Rev. Caatinga*. **2004**, *17*, 52-56.

29. Costa, A.M.D.; Souza, D.P.M.; Cavalcante, T.V.; Araújo, V.L.; Ramos, A.T.; Maruo, V.M. Plantas tóxicas de interesse pecuário em região de Ecótono Amazônia e Cerrado. Parte II: Araguaína, norte do Tocantins. *Acta. Vet. Bras*. **2011**, *5*, 317-324. https://doi.org/10.21708/avb.2011.5.3.2349

30. Helayel, M.A.; Barbosa, F.B.; Carvalho-Júnior, C.P.; Ramos, A.T.; Aguiar-Junior, M.A.; Aguiar, D.M.; Bruns, L.V.; Silva, M.A. Intoxicação natural por *Palicourea marcgravii* (Rubiaceae) em bovinos no Estado do Tocantins. A*rquivos Pesquisa Animal.* **2012**, *1*, 8-12.

31. Schons, S.V.; Lopes, T.V.; Melo, T.L.D.; Lima, J.P.; Riet-Correa, F.; Barros, M.Â.D.B.; Schild, A.L.P. Intoxicações por plantas em ruminantes e equídeos na região central de Rondônia. *Cienc. Rural*. **2012**, *42*, 1257-1263. https://doi.org/10.1590/S0103-84782012005000047

32. Sant'Ana, F.J.F.D.; Reis Junior, J.L.; Freitas Neto, A.P.; Moreira Junior, C.A.; Vulcani, V.A.S.; Rabelo, R.E.; Terra, J.P. Plantas tóxicas para ruminantes do Sudoeste de Goiás. *Cienc. Rural*. **2014**, *44*, 865-871. https://doi.org/10.1590/S0103-84782014000500018

33. Ferreira Junior, J.A.; Novaes, E.D.P.F.; Barbosa, E.F.G.; Nascimento, K.A.; Macêdo, J.T.S.A.; Pedroso, P.M.O. Spontaneous poisoning by *Palicourea marcgravii* in bovine in the Federal District, Brazil. *Ciênc. Animal*. **2021**, *31*, 178-183.

34. Alves, D.A.; Aquino, R.F.; Abreu, C.M.; Lima, V.A.P.; Bispo, C.A.S.; Pereira, G.C. Plantas tóxicas para bovinos em Minas Gerais. *Ciênc. Anim*. **2021**, *31*, 58-66

35. Oliveira, C.M.C.D.; Barbosa, J.D.; Macedo, R.S.; Brito, M.D.F.; Peixoto, P.V.; Tokarnia, C.H. Estudo comparativo da toxidez de *Palicourea juruana* (Rubiaceae) para búfalos e bovinos. *Pesq. Vet. Bras.* **2004**, *24*, 27-30. https://doi.org/10.1590/S0100-736X2004000100007

37. Oliveira, M.D.D.; Riet-Correa, F.; Carvalho, F.K.; Silva, G.B.; Pereira, W.S.; Medeiros, R.M. Indução de resistência à intoxicação por *Palicourea aeneofusca* (Rubiaceae) mediante administração de doses sucessivas não tóxicas. *Pesq. Vet. Bras.* **2013**, *33*, 731-734. https://doi.org/10.1590/S0100-736X2013000600007

38. Barbosa, J.D.; Oliveira, C.M.C.D.; Tokarnia, C.H.; Riet-Correa, F. Comparação da sensibilidade de bovinos e búfalos à intoxicação por *Palicourea marcgravii* (Rubiaceae). *Pesq. Vet. Bras.* **2003**, *23*, 167-172. https://doi.org/10.1590/S0100-736X2003000400005

39. Barbosa, E.D.F.; Cardoso, S.P.; Cabral Filho, S.L.S.; Borges, J.R.J.; Lima, E.M.M.; Riet-Correa, F.; Castro, M.B. Sinais clínicos e patologia da intoxicação crônica experimental de caprinos por *Palicourea marcgravii*. *Pesq. Vet. Bras.* **2015**, *35*, 209-215. https://doi.org/10.1590/S0100-736X2015000300001

41. Serodio, J.J.; Castro, L.T.S.; Morais, T.L.; Cunha, R.D.S.; Sant'Ana, F.J.F.; Juliano, R.S.; Borges, J.R.J.; Fioravanti, M.C.S.; Cunha, P.H.J. Evaluation of the resistance of Nellore, Curraleiro Pe-duro and Pantaneiro cattle breeds by experimental intoxication of *Palicourea marcgravii*. *Toxicon.* **2019**, *168*, 126-130. https://doi.org/10.1016/j.toxicon.2019.07.008

42. Cunha, I.M.; Lessa, D.A.; Carvalho, V.A.; Alencar, N.X.; Teixeira, A.L.; Caldas, A.S.; Souza, G.N.; Helayel, M.J. Clinical and laboratory findings in sheep experimentally poisoned by *Palicourea marcgravii* and the use of magnesium chloride with possible therapeutic effect on poisoning. *Pesq. Vet. Bras.* **2021**, *41*, e06931. https://doi.org/10.1590/1678-5150-PVB-6931

43. Cunha, I.M.; Lessa, D.A.; Carvalho, V.A.; Santos, B.B.; Souza, G.N.; Alencar, N.X.; et al. Serum and immunohistochemical analyses of troponin I in sheep experimentally poisoned with *Palicourea marcgravii*. *Pesq. Vet. Bras.* **2024**, *44*, e07422. https://doi.org/10.1590/1678-5150-PVB-7422

44. Peixoto, T.C.; Nogueira, V.A.; Caldas, S.A.; França, T.N.; Anjos, B.L.; Aragão, A.P.; Peixoto, P.V. Efeito protetor da acetamida em bovinos indica monofluoroacetato como princípio tóxico de *Palicourea marcgravii* (Rubiaceae). *Pesq. Vet. Bras.* **2012**, *32*, 319-328. https://doi.org/10.1590/S0100-736X2012000400008
